# Lnc-SELPLG-2:1 enhanced osteosarcoma oncogenesis via hsa-miR-10a-5p and the BTRC cascade

**DOI:** 10.1186/s12885-022-10040-5

**Published:** 2022-10-05

**Authors:** Shiyuan Li, Ming Zeng, Lin Yang, Jianshao Tan, Jianqi Yang, Hongye Guan, Manyuan Kuang, Jiaying Li

**Affiliations:** Department of Spinal Surgery, the First People’s Hospital of Foshan, North Lingnan Avenue 81, Foshan, 528000 Guangdong China

**Keywords:** lncRNA, Lnc-SELPLG-2:1, Osteosarcoma, Hsa-miR-10a-5p, BTRC

## Abstract

**Background:**

To investigate the potential role of Long Non-coding RNAs (lncRNAs) in the progression of osteosarcoma.

**Methods:**

The candidate lncRNAs were screened with RNA-seq and confirmed with quantitative real-time PCR. Using MTS, transwell assay, and flow cytometric analysis, the effects of overexpressed lnc-SELPLG-2:1 on cell functions were determined. Immunohistochemical staining, fluorescence in situ hybridization, and luciferase reporter assay were used to evaluate the potential mechanism of lnc-SELPLG-2:1 in vivo and in vitro using a tumor model. Moreover, the effects of overexpression of hsa-miR-10a-5p on the functions of SaOS2 cells were determined using functional cell analysis. A response test was used to confirm the mechanism by which lnc-SELPLG-2:1 sponge hsa-miR-10a-5p promotes the expression of BTRC to regulate osteosarcoma.

**Results:**

Lnc-SELPLG-2:1 was highly expressed in osteosarcoma compared to normal cells and bone and marrow samples. Inhibition of lnc-SELPLG-2:1 accelerated cell apoptosis and suppressed cell proliferation, migration, and invasion, whereas lnc-SELPLG-2:1 overexpression had the opposite effect. Moreover, inhibiting lnc-SELPLG-2:1 in an in vivo model decreased tumor size and suppressed the expression of cell migration-related proteins. The prediction, dual luciferase assay, and response test results indicated that hsa-miR-10-5p and BTRC were involved in the lnc-SELPLG-2:1 cascade. Unlike lnc-SELPLG-2:1, hsa-hsa-miR-10a-5p had opposite expression and function. Competitive binding of lnc-SELPLG-2:1 to hsa-hsa-miR-10a-5p prevented BTRC from miRNA-mediated degradation, thereby activating the expression of VIM, MMP9, and MMP2, promoting osteosarcoma cell proliferation, migration, and invasion, and inhibiting apoptosis.

**Conclusion:**

Lnc-SELPLG-2:1 is an oncogenesis activator in osteosarcoma, and its functions are performed via hsa-miR-10a-5p /BTRC cascade.

## Introduction

Osteosarcoma is a common tumor that primarily affects adolescents and children [1]. With the development of multiple techniques, including tumor excision, radiotherapy, and neoadjuvant or adjuvant chemotherapy, the 5-year survival rate for non-metastatic patients has increased to 65% [2]. However, approximately 40–50% of patients eventually develop metastasis, particularly pulmonary metastases [3]. The underlying molecular mechanisms of the disease remain unknown at this time. Therefore, the identification of new candidate molecules will aid in the development of a novel therapeutic approach for osteosarcoma and the improvement of patient clinical outcomes.

Long non-coding RNAs (lncRNAs) are RNAs with a length greater than 200 nt but no or minimal protein-coding ability [4]. LncRNAs can regulate gene expression via various mechanisms, including mRNA splicing, epigenetic silencing, lncRNA-miRNA interaction, lncRNA-mRNA interaction, and lncRNA-protein interaction [5]. Many lncRNAs function as cis-regulators because their expression is highly correlated with that of their adjacent protein-coding genes. However, not all downregulated genes are accompanied by reduced expression of nearby lncRNAs. Often, lncRNA can affect the positive or negative expression of nearby [6]. According to the competing endogenous RNA (ceRNA) hypothesis, some lncRNAs can act as a “sponge” to interact with miRNAs and prevent them from binding to mRNAs [7]. As reported in the study, ceRNAs are present in all organisms in which lncRNAs regulate miRNAs to control gene expression [8, 9]. Given the function of the ceRNA, reports have stated that lncRNAs regulate numerous biological processes, including cell proliferation, apoptosis, migration, invasion, metastasis, and angiogenesis [10–12]. In contrast, lncRNAs can act as oncogenes or tumor suppressors, thereby playing a crucial role in cancer development and carcinogenesis [13, 14]. It has been reported that the expression level of lncRNAs correlates with various types of cancer, including osteosarcoma. Increased expression of the lncRNA FGFR3-AS1 is associated with osteosarcoma, resulting in a large tumor size, advanced Enneking stage, and a poor prognosis [15]. Plasma lncRNA TUG1 is the lncRNA that is used for osteosarcoma detection and dynamic surveillance [16]. Decreased expression of the lncRNA TUSC7 increased osteosarcoma cell proliferation and colony formation in vitro [17]. Inhibition of lncRNA ODRUL can inhibit the proliferation and migration of osteosarcoma cells [18]. Therefore, lncRNAs can be used as molecular biomarkers for the diagnosis of osteosarcoma and as potential therapeutic targets.

In this study, candidate lncRNAs associated with osteosarcoma were evaluated. The correlated miRNAs and genes were predicted and validated in accordance with the ceRNA hypothesis. The functions of lnc-SELPLG-2:1 were uncovered by determining the functions associated with cell proliferation, migration, and invasion. The findings will give insight into functional interactions between lnc-SELPLG-2:1 and hsa-miR-10a-5p, hsa-miR-10a-5p, and BTRC, which are involved in osteosarcoma.

## Material and methods

### Specimen collection

The specimens were retrieved from the departmental archives of the First People’s Hospital of Foshan. These specimens were tissue sections obtained between 2017 and 2020 from patients (*n* = 50) with osteosarcoma who had never received chemotherapy or radiotherapy. Additionally, adjacent normal tissue specimens from one patient obtained 2 cm from the periphery of the cancer site were used as controls. According to the TNM system of the American Joint Committee on Cancer, the patients were diagnosed (AJCC). The First People’s Hospital of Foshan committee approved the experiment (Approval L[2016] No.18, date: December 30, 2016) and all patients signed informed consent forms. All experiments in this study were conducted in accordance with the guidelines in the Declaration of Helsinki.

### Cell culture

ATCC supplied the hFOB, MG63, and SaOS2 cell line strains. All of these cell lines were maintained in a DMEM culture medium supplemented with 10% FBS, 100 U/ml penicillin, and 100 μg/ml streptomycin, per the instructions. They were grown in a humidified incubator containing 5% CO_2_.

### RNA-seq

RNA-seq was conducted similarly to previous studies with a few modifications [19]. Total RNA from the cells was purified using Trizol (Invitrogen, CA, USA) and phenol purification. The RNA integrity was evaluated based on the RIN value using Agilent bioanalyzer 2100 (Agilent, CA, USA). RNA was further purified using the RNA Clean XP Kit (Beckman Coulter, CA, UA), and the DNA residue was removed by the RNase-free DNase Set (QIAGEN, Germany). The quality and concentration of RNA were determined using a NanoDrop 2000 spectrophotometer (Thermo Fisher, USA). rRNA was eliminated using the NEBNext rRNA Depletion kit (NEB, USA). Following the manufacturer’s instructions, 1 μg of total RNA was used to prepare the the library using the VAHTS Total RNA-Seq (H/M/R) Library Prep Kit for Illumina® (NR603–02, Vazyme, Nanjing, China). After RNA fragmentation, the double-stranded cDNA was synthesized.

After performing end-polishing, the cDNA fragments were ligated with adapters. The ligated cDNA was then amplified with universal PCR to generate a sufficient library for sequencing using the Agilent bioanalyzer 2100. The quality of the library was determined. For the RNA-sequencing, Illumina Hiseq 4000 was used. The information was then compiled and annotated with transcript symbols. Subsequently, using R software, the differentially expressed lncRNAs were screened based on fold-change and *p*-value (p-value < 0.05 and fold-change > 2.0).

### GO and pathway analysis

DAVID (Database for Annotation, Visualization, and Integrated Discovery) was used to annotate the potential signaling pathway functions of the corresponding parent genes of the differentially expressed lncRNA [20]. GO functional annotation was then used to predict the functional annotation of the parental genes. A histogram was used to display the GO analysis’s findings. KEGG pathway annotation was used to present the relevant pathways of the differentially expressed lncRNAs’ parent genes. The interaction network between genes and lncRNAs was then predicted and summarized.

### Cell transfection

The lnc-SELPLG-2:1 core sequences were synthesized and cloned into the vector pcDNA3.1(+). The control vector was an empty vector. Genepharma designed and synthesized the siRNA (GCAGCCTGTGTCCACAATA) that targets lnc-SELPLG-2:1 (Shanghai, China). Genepharma was procured for miRNA mimics and inhibitors (Shanghai, China). The vector, siRNA, miRNA mimics, and inhibitor were transfected into cells using lipofectamine 3000 (Thermo Fisher, USA) as previous study [21].

### MTS assay

MTS was conducted following previous research with a few revisions [22]. The cells were at the density of 1 × 10^6^/ml, and 100 μl/well were seeded into 96-well plates. The cells were incubated at 37 °C with 5% CO_2_. After days 1, 2, and 3 of respective treatments and culture, the MTS/PMS mixture (Promega, USA) was added. After 3 h of incubation, the optical density of the cells at 490 nm was measured.

### Cell cycle detection

The detection of the cell cycle was performed in accordance with previous research with a few modifications [22]. PBS was used to harvest and wash the cells. The cells were resuspended with 300 ul of PBS and 700 ul of 100% ethanol. Cells were fixated at − 20 °C overnight. The cell culture supernatant was removed, and the cells were washed with PBS. The cells were resuspended with PI/Rnase and incubated at room temperature for 30 min in the dark. The cell suspension was analyzed using a flow cytometer, and data were analyzed using FLowjo software.

### Cell apoptosis assay

The apoptosis assay was performed in accordance with previous research with a few modifications [22]. Apoptosis was determined using a fluorescence-activated cell sorter stained with APC-Annexin V/7-AAD. The cells were harvested, washed twice with PBS, and resuspended 1 × 10^6^ cells/ml in 100 ul 1× binding buffer (eBioscience, CA). 5 ul APC-Annexin V and 7 ul 7-AAD were added to the cell suspension, which was then incubated for 15 min in the dark at room temperature with 400 ul 1× binding buffer. Samples were analyzed using the FACS Calibur flow cytometer (BD Biosciences, USA) and Cell Quest Research Software.

### Migration and invasion assays

Migration and invasion tests were conducted based on previous research with a few modifications [22]. Utilizing a 24-well plate with 8 μm pore size chamber inserts (Corning, USA), migration and invasion of tumor cells were evaluated. In the upper chamber, 1 × 10^5^ cells were seeded for the migration assay. The membrane was coated with Matrigel (BD Biosciences, USA) to form a matrix barrier for the invasion assay, and then 1 × 10^5^ cells were added to the upper chamber. Each lower chamber received 600 μl of DMEM medium containing 10% FBS. At 37 °C, cells were allowed to migrate for 24 h or invade for 36 h. After incubation, the cells that had migrated through the pore were fixed with 4% paraformaldehyde and stained with 0.1% crystal violet. The cells were then counted and photographed using an inverted IX71 microscope (Olympus, Tokyo, Japan).

### Subcutaneous tumor formation of nude mice

Subcutaneous tumor formation in the nude mice were conducted following previous research with a few modifications [22]. The lnc-SELPLG-2:1 lentivirus was designed and acquired from Genepharma in Shanghai, China. Lentivirus was used to infect SaOS2 cells in order to induce cells with lnc-SELPLG-2:1 inhibition. For the subcutaneous tumor formation assay, cells were used.

A total of 14 nude mice, 5 weeks old, were randomly divided into two groups using a table of random numbers, with 7 animals in each group (NC and si-lnc-SELPLG-2:1 group). The injection was administered following 1 week of feeding the mice. Specifically, mice were rendered unconscious by injecting 45 mg/kg of pentobarbital sodium at a dose of 45 mg/kg weight. Suspension of cells (5 × 10^6^ cells) was injected into the right posterior flank of mice. After 28 days, all mice were sacrificed using CO_2_, and their cancerous tissues were collected and analyzed. One portion of cancerous tissues was removed by sterile surgery and placed in RNase-free saline, while the other portion was fixed with formalin and embedded in paraffin. The animal experiment was approved by the ethical committee of Guangzhou Forevergen Biosciences (Approval No. IACUC-G16053, date: March 24, 2019). All experiments in this study were conducted in accordance with the Basel Declaration, the International Council for Laboratory Animal Science (ICLAS) published ethical guidelines and the American Veterinary Medical Association (AVMA) Guidelines for the Euthanasia of Animals (2020). Moreover, our animal experiments were conducted according to ARRIVE guidelines.

### Immunohistochemical staining

The immunohistochemical staining was followed by several revisions to the previous study [21]. The formalin-fixed and paraffin-embedded tissue were deparaffinated and washed twice with PBS. The peroxidase was inhibited by 3% H_2_O_2_ for 5–20 min and then washed three times with PBS. The tissue was repaired by incubating it in 95 °C water and then washing it with PBS. The tissue was then blocked with 5% BSA and incubated overnight at 4 °C with the primary antibody (MMP2, Cat: ab97779, 1:1000, Abcam; MMP9, Cat: ab38898, 1:500, Abcam; VIM, Cat: 10366–1-AP, 1:3000, Proteintech). After three washes, the tissue was incubated for 1 h at room temperature with a secondary antibody. The sections were developed in freshly prepared diaminobenzidine (DAB) solution, counterstained with hematoxylin, dehydrated through graded ethanol, cleared with xylene, and coverslipped after a thorough PBS wash. Then, using microscopy, the expression levels of the molecules were determined.

### Fluorescence in situ hybridization

Fluorescent in situ hybridization (FISH) was used to detect lnc-SELPLG-2:1 in formalin-fixed and paraffin-embedded human and mouse tumors. Briefly, after dewaxing and rehydration, samples were digested with proteinase K, fixed again in 4% paraformalde, and hybridized with a bersinbio-purchased FAM dye-labeled antisense probe to avoid light (Guangzhou, China). As a negative control, a Sense probe was used. The prehybridization and hybridization processes were conducted at 37 °C for 30 min and 24 h, respectively. DAPI was used and added for staining the section, and the slides were observed under fluorescent microscopy.

### Luciferase reporter assay

The Luciferase reporter assay was performed based on previous research with a few modifications [22]. In both the wild-type and mutant populations, the target sequences of lnc-SELPLG-2:1, the 3’UTR region of AKT2, and BTRC were amplified. Purified amplicons were then cloned into the pmirGLO vector. The sequence was cloned to the Nhel-SalI position. miRNA targets the 3’UTR to regulate gene expression, while lncRNA act as a sponge to bind to miRNA. The cells were co-transfected with miRNA mimics and a reporter vector. The interactions between miRNA, lncRNA, and the target gene were determined based on the relative fluorescent value.

### RT-qPCR

Similar to previous research, we detected RNA expression using RT-qPCR [21]. The levels of LncRNA, miRNA, and mRNA were determined by RT-qPCR. Total RNA was reverse-transcribed using M-MLV Reverse Transcriptase (Promega, USA) per the manufacturer’s instructions. The PCR reaction was conducted using GoTaq qPCR Master Mix (Promega, USA). The PCR amplification was performed using an ABI 7500 system (Applied Biosystem, USA). GAPDH was used as an internal control for the detection of lncRNA and mRNA, while U6 was used for the detection of miRNA. The primer sequences are shown in Table 1.

**Table 1:** The primer sequence used in this study

Primer name	Sequence	Product length
lnc-SELPLG-2:1-F	5′ CAAGGAACTGTGCTGGGATT 3’	246 bp
lnc-SELPLG-2:1-R	5′ AGCAGTAGCGGGATGAGAAA 3’	
GAPDH-F	5′ GAGTCAACGGATTTGGTCGT 3’	185 bp
GAPDH-R	5′ GACAAGCTTCCCGTTCTCAG 3’	
lnc-DDX47–3:1-F	5′ AGGTGGCAGCTTCAGAAAAA 3’	195 bp
lnc-DDX47–3:1-R	5′ AGCATCCTTGGAACATCCTG 3’	
lnc-SRSF2–9:2-F	5′ CCTGGTCGACACCTTGATTT 3’	179 bp
lnc-SRSF2–9:2-R	5′ AAAGTCCCAGGGTCAGTGTG 3’	
lnc-SAMD11–1:1-F	5′ CCAGAAGGTCAGCCGTAGAG 3’	190 bp
lnc-SAMD11–1:1-R	5′ CCACACCAGGAAACCTGTCT 3’	
lnc-PPP1R9B-4:2-F	5′ TGGCCCTCCTGTAAGTATGC 3’	182 bp
lnc-PPP1R9B-4:2-R	5′ TGTGTACCCCTCACCCTCTC 3’	
lnc-ZNF77–165:3-F	5′ GGACTTTCGGAAAAGCCTTC 3’	226 bp
lnc-ZNF77–165:3-R	5′ CCCCAGTAGCTGGGACTACA 3’	
INPPL1-F	5′ CTGGGTGACCTGACCAAGAT 3’	224 bp
INPPL1-R	5′ ATGAAGTCCTTGCGCTGAGT 3’	
HOXA1-F	5′ GGGTGTCCTACTCCCACTCA 3’	162 bp
HOXA1-R	5′ GGACCATGGGAGATGAGAGA 3’	
MAP3K7-F	5′ ACAGTGTTCCCAAGGAGTGG 3’	196 bp
MAP3K7-R	5′ AACTTCAGGTGCCATCCAAG 3’	
BTRC-F	5′ TGTCATACCTGGATGCCAAA 3’	163 bp
BTRC-R	5′ TACTGTCCCCATCCTCTTCG 3’	
U6-F	5′ CGCTTCGGCAGCACATATAC 3’	83 bp
U6-R	5′ CGAATTTGCGTGTCATCCTTG 3’	

### Statistical analysis

All experiment was performed at least 3 times. When data corresponded to a normal distribution, comparisons were performed using independent t-tests, one-way and two-way ANOVA was applied if data did not correspond to a normal distribution. The significance level was established as *P* < 0.05. For the statistical analysis, GraphPad Prism 7 software was used.

## Results

### RNA-seq revealed lnc-SELPLG-2:1 can be a candidate lncRNA involved in osteosarcoma

**Fig. 1:**
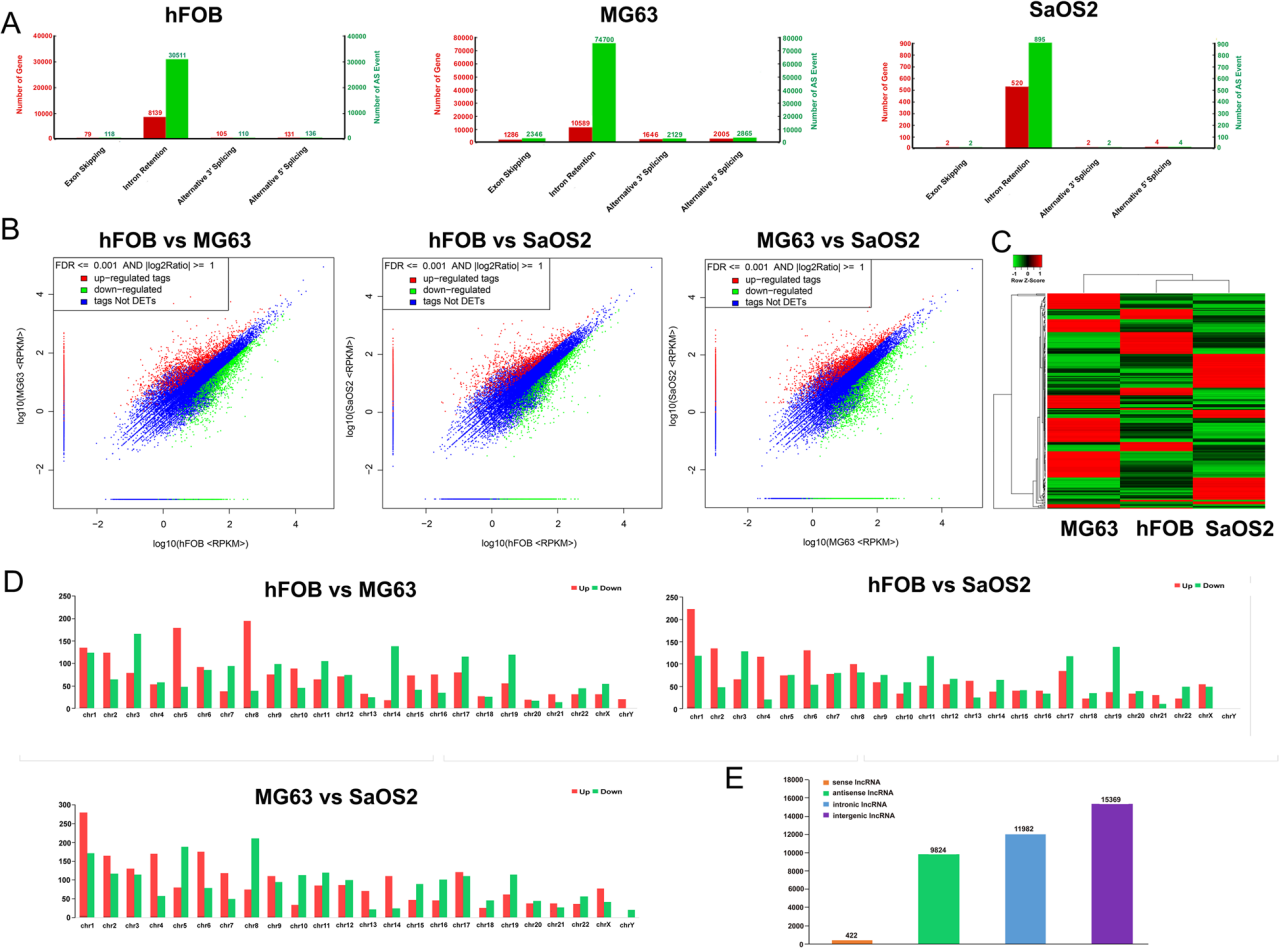
RNA-seq analysis of hFOB, MG63, and SaOS2 cells for lnc-RNA expression profiling. **A** lncRNA alternative splicing in hFOB, MG63, and SaOS2 cells. **B** Dot plot of the gene expression level in three comparisons: hFOB vs. MG63, hFOB vs. SaOS2, and MG63 vs. SaOS2. **C** Cluster diagram illustrating the differential lncRNA expression profiles of hFOB, MG63, and SaOS2 cells (*P* < 0.01, FDR < 0.01, |log2 Ratio FC| > 1). **D** Differential chromosomal lncRNA chromosome distribution for hFOB vs. MG63, hFOB vs. SaOS2, and MG63 vs. SaOS2. **E** Categories of lncRNAs

The purpose of the lncRNA sequencing was to validate the candidate lncRNAs implicated in osteosarcoma. The cell lines MG63 and SaOS2 were used in the study, with hFOB as the control. Alternative splicing was later identified using the sequencing profiles of the cells. According to the results, the predominant alternative splicing pattern for the RNAs in each cell was intron retention, which was more significant than exon skipping, alternative 5′ splicing, and alternative 3′ splicing (Fig. 1A). Figure 1B summarizes the dot plots of the gene expression profiles for the comparisons of hFOB vs. MG63, hFOB vs. SaOS2, and MG63 vs. SaOS2. Regardless of the comparisons of hFOB vs. MG63, hFOB vs. SaOS, and MG63 vs. SaOS2, many lncRNAs differed. Figure 1C depicts the cluster diagram of differential lncRNAs in the hFOB, SaOS2, and MG63. According to a summary of the results, there were 252 upregulated, and 612 downregulated common lncRNAs within hFOB versus MG63 and hFOB versus SaOS2. Figure 1D summarizes the chromosome distribution of differential lncRNAs between hFOB and MG63, hFOB and SaOS2, and MG63 and SaOS2. In terms of chromosome distribution, there is no significant difference between the three comparisons, hFOB versus MG63, hFOB versus SaOS2, and MG63 versus SaOS2. However, in osteosarcoma cells, high expressed lncRNAs were identified on chromosome 2, while low expressed lncRNAs were identified on chromosomes 3, 14, 17, and 19. Most of the differentially expressed lncRNAs within the cells were intervening lncRNAs, secondary intrinsic lncRNAs, and antisense lncRNAs, while there were significantly fewer sense lncRNAs (Fig. 1E). Functional analysis of the lncRNAs was conducted to gain a deeper understanding of their functions.

**Fig. 2:**
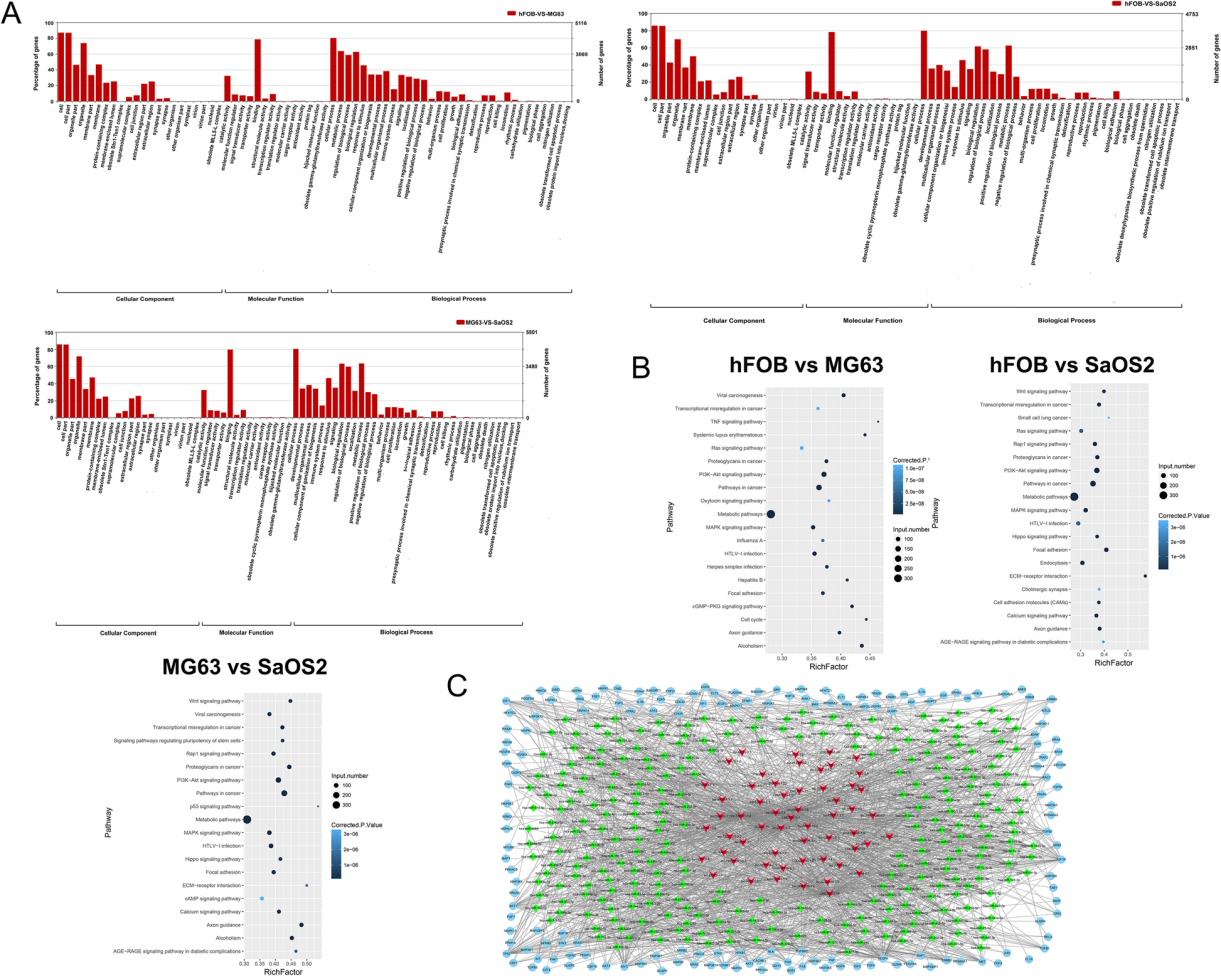
Analysis of the function of differential lncRNAs in hFOB, MG63 and SaOS2 cells. **A** GO analysis of the functions of the respective lncRNA-derived genes. **B** KEGG pathway analysis of the genes from which the corresponding lncRNAs originated. **C** MAPK pathway-involved lncRNA-miRNA-mRNA network of common lncRNAs in hFOB, MG63, and SaOS2 cells. The red V-shape represents lncRNA, the green square represents miRNA, and the blue circle represents mRNA

The originated genes of the significantly expressed lncRNAs in the three comparisons were summarized and analyzed using GO terms (Fig. 2A). Most of the significantly originated genes of the corresponding expressed lncRNAs were involved in cellular components and biological processes, whereas only a few were involved in molecular functions. Similarly, originated genes of significantly expressed lncRNAs in the three comparisons were analyzed by KEGG for functional annotation (Fig. 2B). The common pathways in the three comparisons included cancer pathways, metabolic pathways, the PI3K-AKT pathway, and the MAPK pathway. The lncRNA, miRNA, and mRNA-based network of the MAPK pathway containing lncRNA, miRNA, and mRNA was created due to the significance of the MAPK pathway in various types of cancer and its role in regulating essential processes, such as metastasis, cell proliferation, and apoptosis (Fig. 2C). Two lncRNAs exhibited differential expression in the cells, as shown in Table 2, and 4 lncRNAs in the regulatory network exhibited the potential for performing essential functions. The MAPK pathway-involved lncRNAs were selected for further study.

**Table 2:** The expression of selected lncRNA in sequencing

geneID	hFOB-RPKM	MG63-RPKM	SaOS2-RPKM	Diff	length
lnc-SELPLG-2:1	1.953728683	544.0501308	650.4964421	Up	751
lnc-DDX47–3:1	808.5941109	181.1645482	0.974278704	Down	2740
lnc-ZNF77–165:3	44.59605059	10.30463314	5.847806463	Down	1826
lnc-SRSF2–9:2	12.47396592	39.03985956	41.13506156	Up	1882
lnc-SAMD11–1:1	5.062808916	22.37822589	40.64670691	Up	3034
lnc-PPP1R9B-4:2	39.48939046	118.9681518	166.0469256	Up	836

**Fig. 3:**
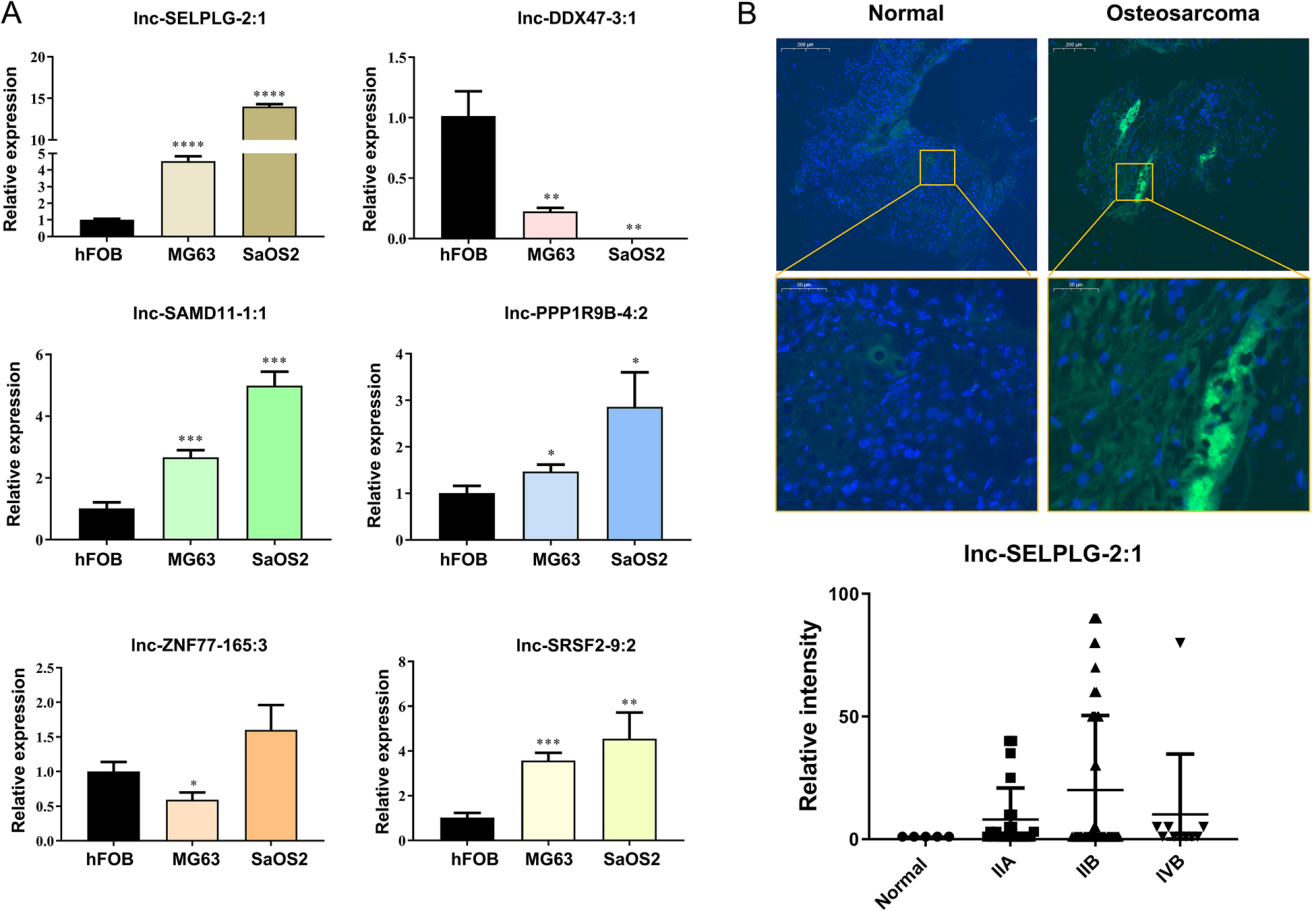
Validation of the candidate lncRNAs. **A** The candidate lncRNAs in hFOB, MG63, and SaOS2 cells were validated by RT-qPCR (*P < 0.05; **P < 0.01; ***P < 0.001; ****P < 0.0001). **B** Fluorescence in situ hybridization revealed a high level of lnc-SELPLG-2:1 expression in the tissues of osteosarcoma. And RT-qPCR results also indicated that the expression level of lnc-SELPLG-2:1 is higher in the tumorous tissues of osteosarcoma patients in phases IIA, IIB, and IVB

In order to determine which lncRNA was involved in osteosarcoma, lncRNAs including lnc-SELPLG-2:1, lnc-DDX47–3:1, lnc-ZNF77–165:3, lnc-SRSF2–9:2, lnc-SAMD11–1:1, and lnc-PPP1R9B-4:2 were detected by RT-qPCR to determine if their sequences were consistent with sequencing results. Initially, lnc-ZNF77–165:3 was downregulated in MG63 cells but upregulated in SaSO2 cells, which was inconsistent with the sequencing results and therefore discarded. Lnc-DDX47–3:1 exhibited downregulated expression in MG63 and SaOS2 cells, whereas the rest of the lncRNAs exhibited upregulated expression in MG63 and SaOS2 cells relative to hFOB cells, as shown by lncRNA sequencing (Fig. 3A). Therefore, we chose lnc-SELPLG-2:1, which showed the highest expression level in MG63 and SaOS2 cells, for further study. To determine the level of lnc-SELPLG-2:1, we collected tumorous and normal tissues from osteosarcoma patients and performed FISH. As shown in the results, the lnc-SELPLG-2:1 will be specifically stained with a fluorescent green signal. It is expressed within the cytoplasm of the tumorous tissue. The level of lncRNA was significantly elevated in tumorous tissue compared to adjacent normal tissue (Fig. 3B). The relative intensity also indicated that the expression level of lnc-SELPLG-2:1 is higher in the tumorous tissues of osteosarcoma patients in phases IIA, IIB, and IVB (Fig. 3B). Therefore, it is confirmed that lnc-SELPLG-2:1 expression is upregulated and may be implicated in osteosarcoma patients.

### Lnc-SELPLG-2:1 promoted proliferation, cell invasion, and migration while inhibited apoptosis

**Fig. 4:**
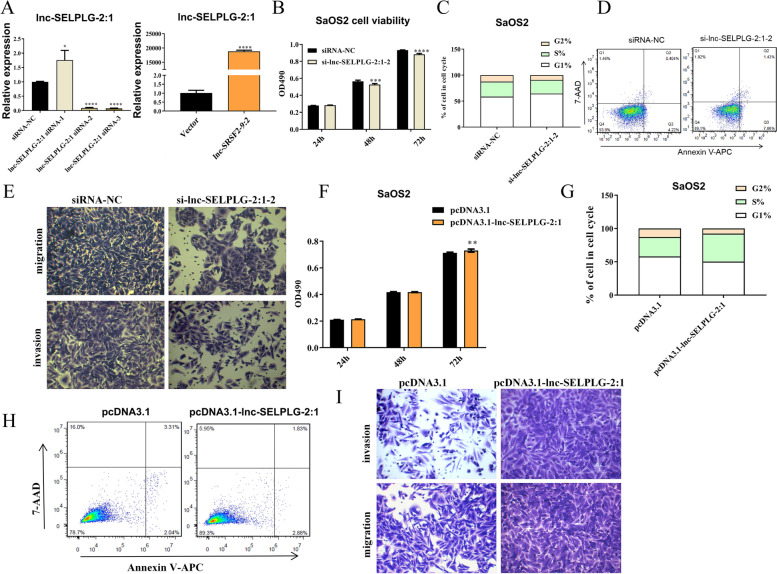
Inhibition and overexpression of lnc-SELPLG-2:1 to effect of cell functions of SaOS2 cells. **A** RT-qPCR validation of the performance of siRNA and lnc-SELPLG-2:1 overexpression vector. **B** lnc-SELPLG-2:1 inhibition decreased cell viability as measured by the MTS assay. **C** Increased cells were retained at G1 phase when lnc-SELPLG-2:1 was inhibited via flow cytometry of cell cycle detection. **D** Increased cell apoptosis percentage was observed when lnc-SELPLG-2:1 was inhibited via flow cytometry of apoptosis assay. **E** Capacity of cell migration and invasion was inhibited when lnc-SELPLG-2:1 was inhibited via transwell assay. **F** Cell viability was increased when lnc-SELPLG-2:1 was overexpressed via MTS assay. **G** Flow cytometry of cell cycle detection revealed that when lnc-SELPLG-2:1 was overexpressed, more cells were retained in the S phase. **H** Decreased cell apoptosis percentage was observed when lnc-SELPLG-2:1 was overexpressed via flow cytometry of apoptosis assay. **I** Cell migration and invasion capacity was enhanced when lnc-SELPLG-2:1 was overexpressed via transwell assay. (*P < 0.05; **P < 0.01; ***P < 0.001; ****P < 0.0001)

In order to verify the functions of lnc-SELPLG-2:1, an overexpression vector and lncRNA-specific siRNA were designed. Subsequently, using RT-qPCR, the RNA level of lnc-SELPLG-2:1 was determined following transfection into SaOS2 cells. According to the results, cells transfected with an overexpression vector had a higher concentration of lnc-SELPLG-2:1 than cells transfected with an empty vector (Fig. 4A). For lnc-SELPLG-2:1 inhibition, specific siRNAs were designed. According to the RT-qPCR results, lnc-SELPLG-2:1-siRNA-1 did not inhibit the expression of lnc-SELPLG-2:1, whereas lnc-SELPLG-2:1-siRNA-2 and lnc-SELPLG-2:1-siRNA-3 successfully inhibited lncRNA expression, indicating that both siRNAs can be used in future research. Since both siRNAs demonstrated comparable performance, we chose lnc-SELPLG-2:1-siRNA-2 for further research.

The viability of the SaOS2 cells transfected with siRNA was determined. For 24 h, it did not show any significant difference in cell viability. However, transfection with si-lnc-SELPLG-2:1-siRNA-2 for 48 h and 72 h significantly reduced cell proliferation compared to the siRNA-NC group (Fig. 4B). After inhibiting siRNA, cells were also subjected to flow cytometry assays to determine the change in cell apoptosis and cell cycle. When lnc-SELPLG-2:1 was inhibited, apoptotic cells increased dramatically (Fig. 4D), and a greater proportion of cells remained in the G1 phase while fewer cells were in the S phase in the si-lnc-SELPLG-2:1-siRNA-2 group compared to the siRNA-NC group (Fig. 4C). When lnc-SELPLG-2:1 was inhibited, these indicators suggested that cell proliferation was inhibited and apoptosis was induced. Cell transwell assays were conducted to detect cell migration and invasion. For both assays, it was demonstrated that the capacity for cell migration and invasion was inhibited, as the number of migrated and invasive cells were significantly lower in the lnc-SELPLG-2:1 inhibition group than in the siRNA-NC group (Fig. 4E).

SaOS2 cells were transfected with the overexpression vector, and the empty vector pcDNA3.1 served as the control. It produced opposite outcomes to those of lnc-SELPLG-2:1 inhibition. Furthermore, 72 h after lnc-SELPLG-2:1 overexpression, the cell proliferation was enhanced (Fig. 4F). When lnc-SELPLG-2:1 was overexpressed, cell apoptosis was inhibited, and the percentage of apoptotic cells was significantly reduced (Fig. 4H). When lnc-SELPLG-2:1 was overexpressed, cell cycle analysis revealed that a greater proportion of cells were retained in the S phase, indicating that cell proliferation was enhanced (Fig. 4G). Cell transwell assays demonstrated that lnc-SELPLG-2:1 overexpression significantly enhanced cell migration and invasion (Fig. 4I). Therefore, the evidence demonstrated that lnc-SELPLG-2:1 levels promote cell proliferation. It inhibits cell apoptosis and increases the ability of cells to invade and migrate.

**Fig. 5:**
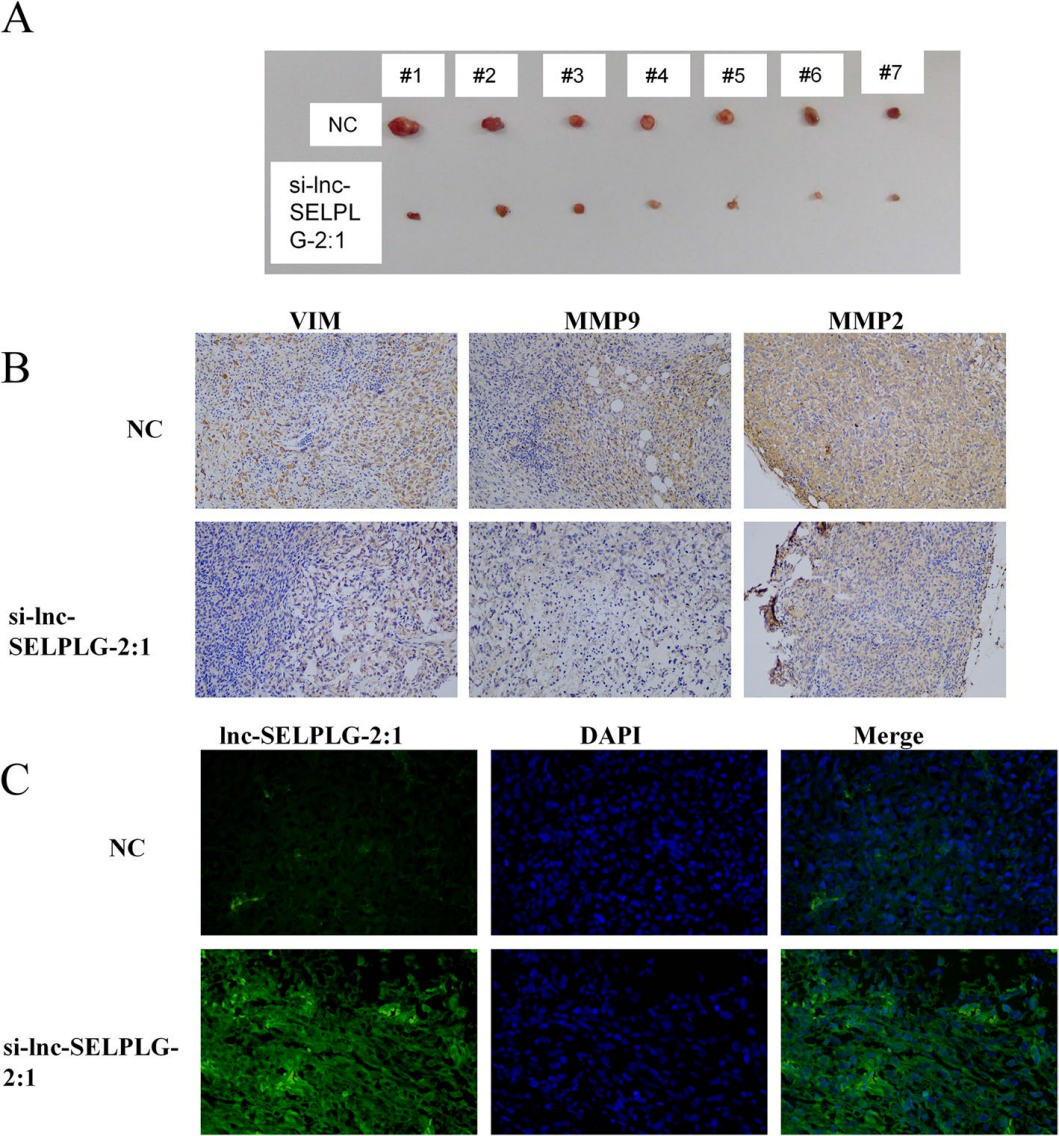
In vivo tumor formation assay to test the functions of lnc-SELPLG-2:1. **A** The size of the tumors in cells treated with sh-NC or sh-lnc-SELPLG-2:1. **B** Immunohistochemical staining of tumor tissue to determine the protein levels of VIM, MMP2, and MMP9. **C** The expression of lnc-SELPLG-2:1 in tumor tissue was confirmed by fluorescence in situ hybridization

In order to confirm the functions of lnc-SELPLG-2:1 further, a tumor formation assay was conducted. After mice were sacrificed, tumor tissues were collected. Furthermore, these results indicated that inhibition of lnc-SELPL-2:1 was associated with a smaller tumor size than sh-NC treatment (Fig. 5A). Therefore, lnc-SELPLG-2:1 can be correlated with cell proliferation, and inhibiting lnc-SELPLG-2:1 expression will inhibit the proliferation of tumorous cells. The expression of the genes involved in cell proliferation and migration was determined by immunohistochemical staining. The expression of these genes, VIM, MMP9, and MMP2, decreased when lnc-SELPLG-2:1 was inhibited (Fig. 5B). As confirmed by FISH, the level of lnc-SELPLG-2:1 was reduced in the tumor after treatment with sh-lnc-SELPLG-2:1, demonstrating that the sh-RNA was effective (Fig. 5C). Therefore, it is consistent with the evidence that inhibition of lnc-SELPLG-2:1 expression suppressed cell proliferation and migration.

### Lnc-SELPLG-2:1 was regulated expression of hsa-miR-10a-5p and BTRC

**Fig. 6:**
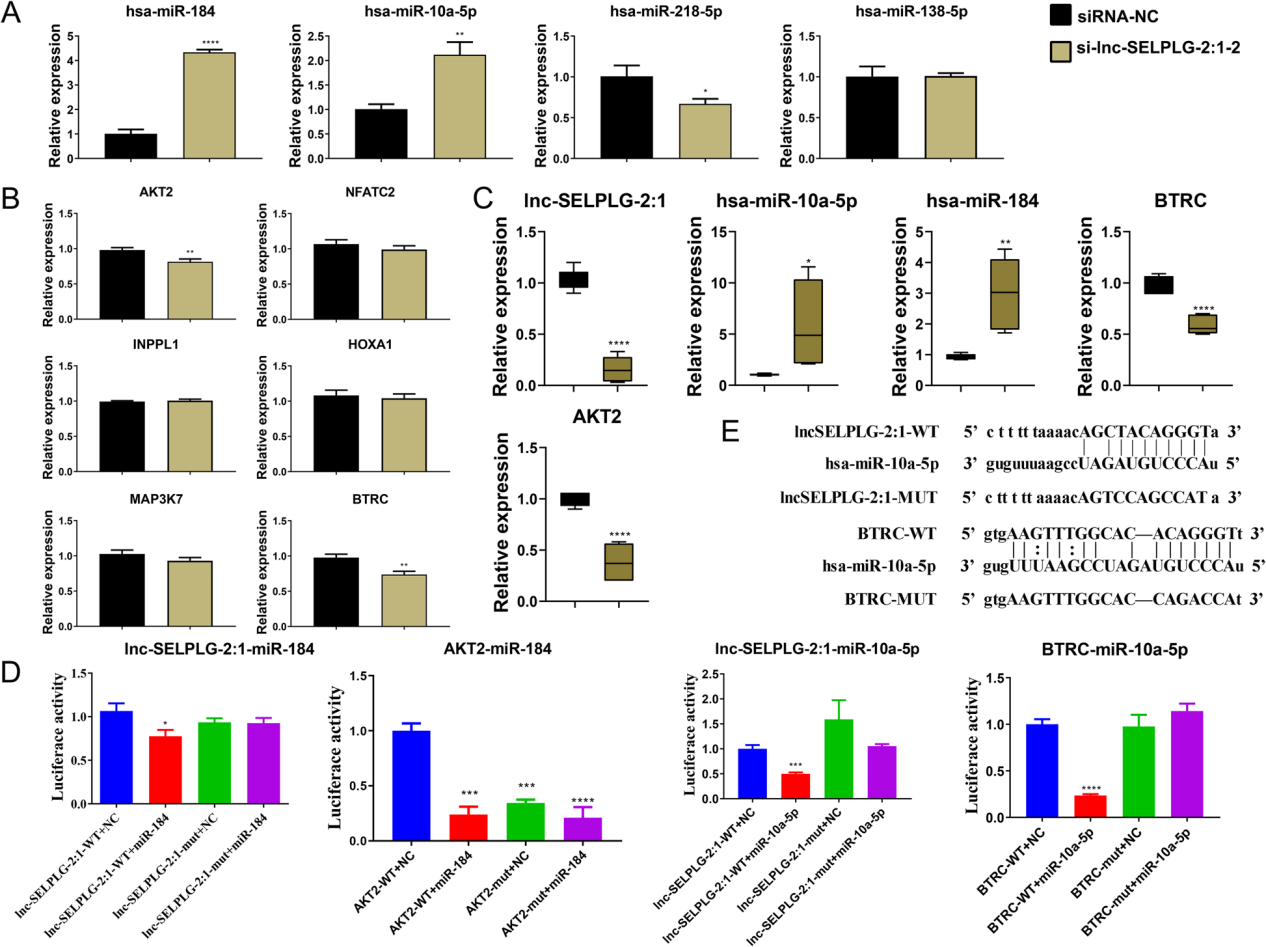
Validation of the target miRNAs and mRNAs of lnc-SELPLG-2:1. **A** RT-qPCR in validation of the target miRNAs of lnc-SELPLG-2:1 in SaOS2 cells. **B** Validation of target mRNA in SaOS2 cells. **C** Validation of the target microRNAs, hsa-hsa-miR-10a-5p and hsa-hsa-miR-184, and messenger RNAs, AKT2 and BTRC, in tumor tissues formed in vivo. **D** Dual-luciferase assay for validating lncRNA-miRNA and miRNA-mRNA interactions. **E** hsa-miR-10a-5p targets lnc-SELPLG-2:1 and BTRC for their binding sites and corresponding mutant region. (*P < 0.05; **P < 0.01; ***P < 0.001; ****P < 0.0001)

In order to investigate the potential mechanism of lnc-SELPLG-2:1, its predicted miRNA targets were analyzed. Four miRNAs for further validation in SaOS2 cells: hsa-miR-184, hsa-miR-10a-5p, hsa-miR-218-5p, and hsa-miR-138-5p. The cells were treated with si-lnc-SELPLG-2:1–2, and the expression levels of the miRNAs were measured and compared to those of siRNA-NC-treated SaOS2 cells. According to the results, the expression of hsa-miR-184 and hsa-miR-10a-5p was upregulated, while that of hsa-miR-218-5p was downregulated, and hsa-miR-138-5p did not exhibit any significant change (Fig. 6A). Based on the ceRNA principle, downregulated expression of lnc-SELPLG-2:1 should induce up-regulation of its targets. Consequently, hsa-miR-184 and hsa-miR-10a-5p can be lnc-SELPLG-2:1 targets.

Similarly, we predicted the targets of hsa-miR-184 and hsa-miR-10a-5p and used RT-qPCR to validate these predictions. AKT2 and BTRC were significantly downregulated when lnc-SELPLG-2:1 was downregulated, whereas the expression of other genes was not significantly altered (Fig. 6B). As miRNA typically inhibits downstream target genes, downregulated expression of lnc-SELPLG-2:1 induced upregulated expression of the target miRNAs, resulting in downregulated expression of downstream genes. Therefore, AKT2 and BTRC can serve as potential targets for lnc-SELPLG-2:1.

We confirmed the hypothesis regarding the tumorous tissues generated by the in vivo tumor formation assay using RT-qPCR. lnc-SELPLG-2:1 was detected, along with its targets hsa-miR-10a-5p, hsa-miR-184, BTRC, and AKT2. lnc-SELPLG-2:1 was downregulated and expressed under the treatment of si-lnc-SELPLG-2:1, hsa-miR-10a-5p, and hsa-miR-184 were upregulated expressed, and BTRC and AKT2 were downregulated expressed. The results were consistent with those conducted in SaOS2 cells (Fig. 6C). The luciferase reporter was developed to validate the interaction between lncRNA, miRNA, and genes. In the lnc-SELPLG-2:1 reporter assay, the targeting relationship between lnc-SELPLG-2:1 and miR-184, hsa-miR-10a-5p, AKT2, and miR-184, and BTRC and hsa-miR-10a-5p was determined. For miR-184 and hsa-miR-10a-5p, the induction of the miRNAs mimics led to a significant decrease in the luciferase activity in the lnc-SELPLG-2:1 wild-type reporter, but no change in the lnc-SELPLG-2:1 mutant reporter, indicating that there are valid interactions between lnc-SELPLG-2:1 and miR-184, and lnc-SELPLG-2:1 and hsa-miR-10a-5p. Induction of miR-184 mimics decreases luciferase activity in AKT2-wild-type and AKT2-mutant reporters, indicating that miR-184 does not target the 3’UTR of AKT2 to perform its function. For the BTRC reporter, the induction of hsa-miR-10a-5p mimics significantly decreased the luciferase activity of the BTRC wild-type reporter but had no effect on the BTRC mutant reporter, indicating that hsa-miR-10a-5p targets the 3′ UTR to perform its functions (Fig. 6D). Figure 6E represents the WT and MUT sequences designed for lnc-SELPLG-2:1 and BTRC based on their binding sites with hsa-miR-10a-5p. The cascade of lnc-SELPLG-2:1, hsa-miR-10a-5p and BTRC was validated based on the evidence and the ceRNA principle.

### Hsa-miR-10a-5p regulates cell proliferation, apoptosis, migration, and invasion of osteosarcoma

**Fig. 7:**
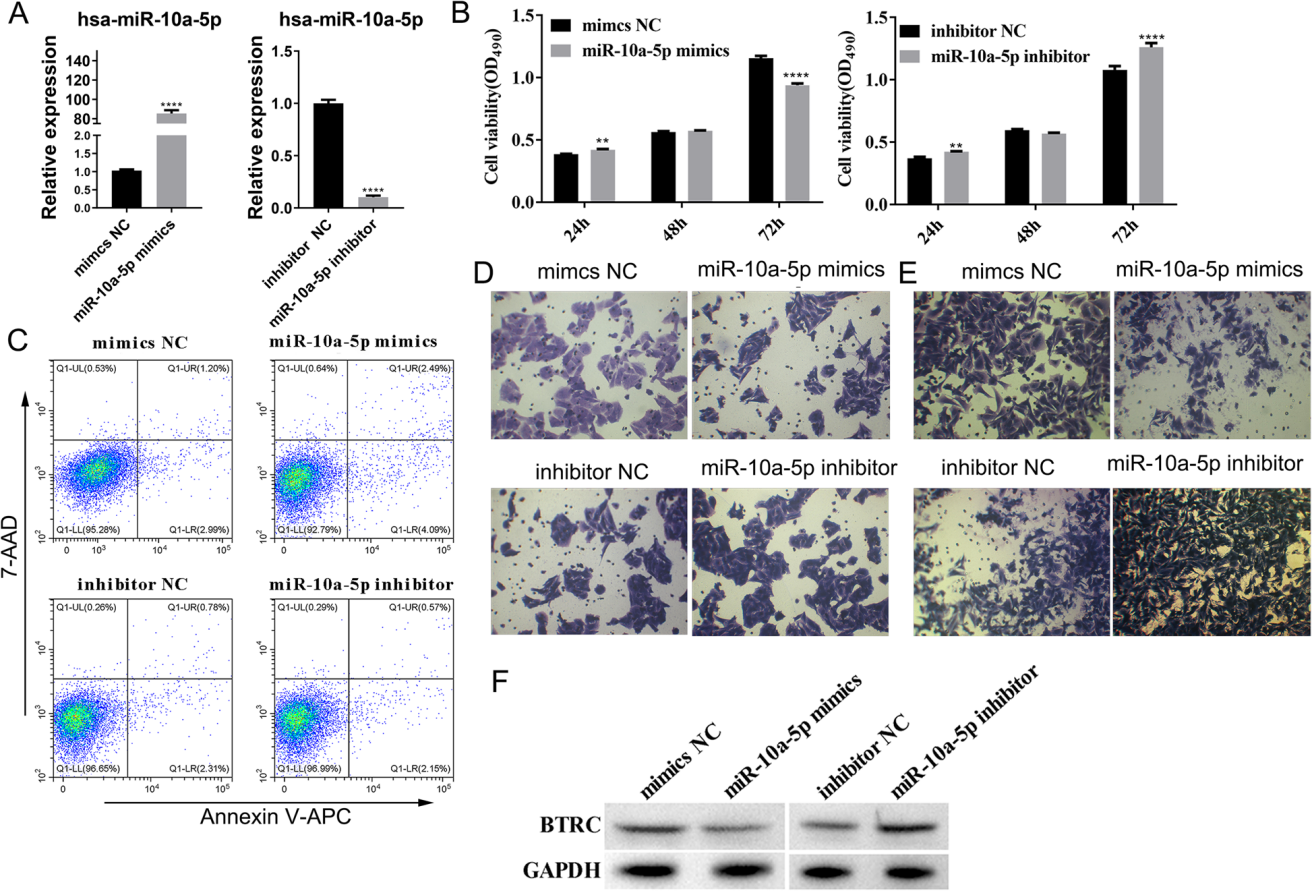
Validation of the functions of hsa-miR-10a-5p. **A** Verify the performance of hsa-miR-10a-5p mimics and inhibitors using RT-qPCR. **B** MTS was used to determine cell viability after SaOS2 cells were transfected with mimics or inhibitors of hsa-miR-10a-5p. **C** Flow cytometry of apoptosis assay was used to test cell apoptosis assay after SaOS2 cells were transfected with mimics or inhibitors of hsa-miR-10a-5p. **D** A transwell assay was used to examine cell migration after SaOS2 cells were transfected with hsa-miR-10a-5p mimics or inhibitors. **E** A transwell assay was used to assess cell invasion after SaOS2 cells were transfected with hsa-miR-10a-5p mimics or inhibitors. **F** hsa-miR-10a-5p negatively regulated the BTRC protein expression level. (*P < 0.05; **P < 0.01; ***P < 0.001; ****P < 0.0001)

For the functional validation of hsa-miR-10a-5p, SaOS2 cells were transfected with miRNA mimics and an inhibitor of hsa-miR-10a-5p to determine the effect on cell proliferation, apoptosis, migration, and invasion. RT-qPCR was used to determine the expression of hsa-miR-10a-5p in SaOS2 cells transfected with hsa-miR-10a-5p mimics and inhibitors. As demonstrated by the results, mimics can induce an 80-fold increase in hsa-miR-10a-5p expression, whereas inhibitors resulted in a 90% decrease in miRNA expression (Fig. 7A). Therefore, the mimics and inhibitors were successfully transfected into the cells. For this condition, cell proliferation was observed; however, 24 h and 48 h after transfection with mimics and inhibitors, there were no significant changes. However, 72 h after transfection, the cell viability was significantly lower in the hsa-miR-10a-5p mimics group than in the mimics NC group, whereas it was higher in the hsa-miR-10a-5p inhibitor group than in the inhibitor NC group (Fig. 7B). For the cell apoptosis assay, the percentage of cell apoptosis was higher in the hsa-miR-10a-5p mimics group and lower in the hsa-miR-10a-5p inhibitors group compared to the NC group (Fig. 7C). We investigated the relationship between hsa-miR-10a-5p expression level along with cell migration and invasion capacity and conducted Transwell assays. Overexpression of hsa-miR-10a-5p by mimics inhibited cell migration and invasion, whereas inhibition of hsa-miR-10a-5p led to increased cell migration and invasion (Fig. 7D&E). After transfection of hsa-miR-10a-5p mimics and inhibitors in SaOS2 cells, the expression of BTRC was detected by western blot. The results showed that the expression of BTRC protein was inhibited by hsa-miR-10a-5p, while the expression of BTRC protein was increased after hsa-miR-10a-5p was inhibited (Fig. 7F). As we have previously learned, downregulated lnc-SELPLG-2:1 expression induced upregulated hsa-miR-10a-5p expression, thereby inhibiting the downregulation of BTRC expression and affecting cell proliferation, migration, and invasion. These results are consistent with previous findings which state that mimic increased hsa-miR-10a-5p; it was concluded that lnc-SELPLG-2:1 may influence the proliferation, migration, and invasion of osteosarcoma cells via hsa-miR-10a-5p /BTRC.

### Lnc-SELPLG-2:1 sponge hsa-miR-10a-5p to promote the expression of BTRC to regulate osteosarcoma

**Fig. 8:**
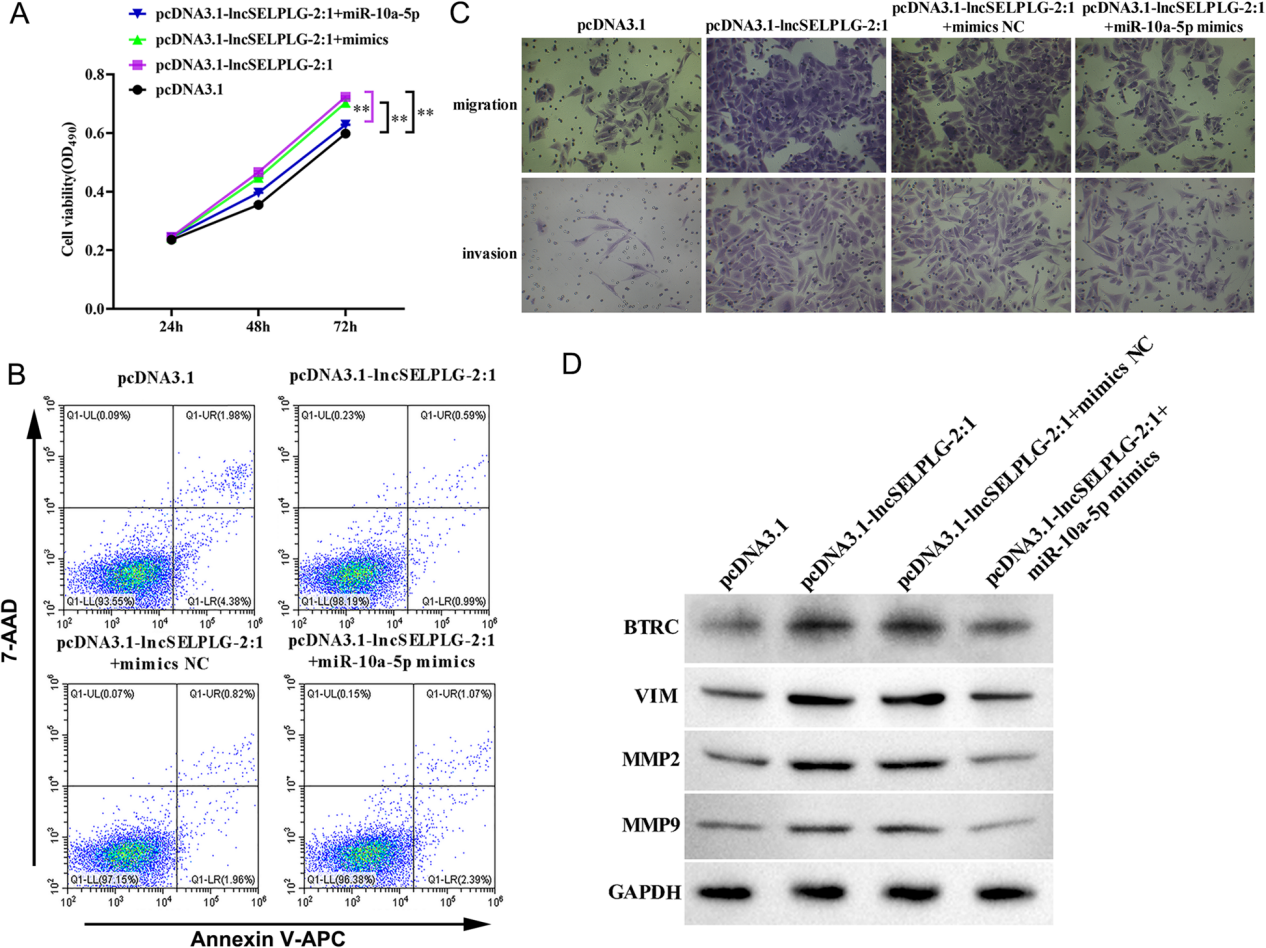
Lnc-SELPLG-2:1 sponge hsa-miR-10a-5p to promote the expression of BTRC to regulate osteosarcoma. **A** MTS was used for cell viability testing. **B** Flow cytometry assay for apoptosis. **C** Transwell assay was used to test cell migration and invasion. **D** BTRC, VIM, MMP2, and MMP9 protein expression was analyzed in SaOS2 cells co-transfected with hsa-miR-10a-5p mimics, pcDNA3.1-lncSELPLG-2:1, or their respective NCs. (*P < 0.05; **P < 0.01; ***P < 0.001; ****P < 0.0001)

As shown in Fig. 8A and C, the proliferation, migration, and invasion of osteosarcoma cells in the pcDNA3.1-lncSELPLG-2:1 group were significantly higher than in the corresponding pcDNA3.1 group, while apoptosis had the opposite effect (Fig. 8B). These results are consistent with those of previous research. However, co-transfection of hsa-miR-10a-5p mimic and lnc-SELPLG-2:1 expression vector revealed that hsa-miR-10a-5p significantly inhibited the cell proliferation, migration, and invasion induced by lnc-SELPLG-2:1, whereas apoptosis had the opposite effect. The co-transfection of mimics NC and pcDNA3.1-lnc-SELPLG-2:1 demonstrated that the ability of SaOS2 cells to proliferate, migrate, and invade was unaffected by the mimics NC, and apoptosis was also unaffected. Furthermore, the BTRC, VIM, MMP2, and MMP9 protein expression trends in all groups were consistent with the cell function trends. These results demonstrated that lnc-SELPLG-2:1 regulates osteosarcoma by competitively binding with hsa-miR-10a-5p to regulate BTRC expression.

## Discussion

In this study, lnc-SELPLG-2:1 was identified as a potential regulator of osteosarcoma processes, including cell proliferation, apoptosis, migration, and invasion. Based on the results and validation, hsa-miR-10a-5p and BTRC were validated as one of the candidate cascades for lnc-SELPLG-2:1 to regulate the processes.

Currently there is no relevant report regarding the functions of lnc-SELPLG-2:1. This gene is an inflammatory molecule related to immune cell differentiation and leukocyte mobilization for its neighbor gene, SELPLG/PSGL-1 [23]. Cell differentiation was impaired in myeloid cells lacking SELPLG [24, 25]. The presence of SELPLG cells was associated with favorable TNM staging and decreased lymph node metastasis [26]. In a study of colorectal cancer specimens, lnc-SELPLG-2:1 expression was increased based on our findings. As lncRNA may affect the expression of their neighboring genes positively or negatively, the gene SELPLG may be impacted, resulting in osteosarcoma processes associated with a poor prognosis. However, it requires additional validation. In our study, we focused on the function and regulation cascade of lnc-SELPLG-2:1 and demonstrated that the lncRNA was upregulated in osteosarcoma specimens, blocking the lncRNA inhibits cell proliferation, migration, and invasion. Therefore, we hypothesized that lnc-SELPLG-2:1 may serve as an osteosarcoma tumor activator.

Based on the ceRNA principle and PCR validation, hsa-miR-10a-5p and hsa-miR-184 were predicted as the targets of lnc-SELPLG-2:1. Furthermore, hsa-miR-10a-5p is a commonly found miRNA that is expressed in various cancers, and reports state that it exhibited high expression osteosarcoma and was associated with shorter overall survival [27]. In other types of cancer, miR-10a was reported to increase in NSCLC and led to tumor node and lymph node metastasis. Moreover, it promoted cell proliferation, migration, and invasion by targeting PTEN [28]. miR-10a was increased in glioma and promoted cell migration and invasion by regulation Eph48 [29]. However, it showed a significant decrease in breast cancer and was partially regulated through retinoic acid [30]. Therefore, hsa-miR-10a-5p does not always present high expression in all types of cancers, indicating that there are differences in the regulation mechanism of the miRNA in different types of cancers.

In our study, since lnc-SELPLG-2:1 was upregulated expressed, it can be predicted that hsa-miR-10a-5p can be downregulated expressed under the regulation of lnc-SELPLG-2:1 overexpression. Therefore, it is not consistent with the previous report. We hypothesized that it can be indicated that hsa-miR-10a-5p was not regulated under lnc-SELPLG-2:1 but can also be regulated by other factors. In other words, it is the only regulation cascade for converting lnc-SELPLG-2:1 to hsa-miR-10a-5p and vice versa. Studies have also reported on the function of hsa-miR-184. Yin et al. indicated that hsa-miR-184 promotes cell proliferation and invasion [31]. Du et al. indicated that hsa-miR-184 can be involved in the Wnt/beta-catenin signaling pathway and that the inhibition of hsa-miR-184 might reduce the tumor volume of osteosarcoma [32]. Those studies provide opposite evidence to our results that lnc-SELPLG-2:1 is higher expressed in osteosarcoma and is correlated to enhanced cell proliferation, migration, and invasion. High expression of lnc-SELPLG-2:1 led to lower expression of hsa-miR-184, which is inconsistent with the functions described by the previous studies above. Therefore, lnc-SELPLG-2:1 might not predominately regulate hsa-miR-184 to proceed with its functions but by regulating other targets instead.

Furthermore, we sought to investigate the targets of hsa-miR-10a and target the candidate gene BTRC. As mentioned in a previous study, BTRC showed significantly downregulated expression in osteosarcoma [33]. BTRC can interact with TSPAN15 and promotes esophageal squamous cell carcinoma metastasis by activating NF-kB signaling [34]. According to our findings, osteosarcoma specimens with upregulated lnc-SELPLG-2:1 and downregulated hsa-miR-10a-5p exhibited upregulated BTRC expression. Therefore, it appears that neither hsa-miR-10-5p nor the BTRC results from the previous studies were consistent with our findings. Furthermore, we have examined the functions of hsa-miR-10a-5p individually through overexpression and inhibition. It was demonstrated that the downregulation of hsa-miR-10a-5p increased SaOS2 cell proliferation, migration, and invasion.

Consequently, the functions of hsa-miR-10a-5p can be associated with enhanced oncogenesis processes. Since we did not individually test the functions of BTRC and determine the expression levels of hsa-miR-10a-5p and BTRC in specimens from osteosarcoma patients, it is difficult to conclude whether the expression levels of both molecules can be consistent between in vitro cell assays and clinical specimens. The potential regulation cascade of lnc-SELPLG-2:1 that correlates with hsa-miR-10a-5p and BTRC may be a way the lncRNA performs its functions as suggested here.

However, it may not be the only option. Both of these molecules, like hsa-miR-10a-5p and BTRC, can be regulated by other molecules. The actual regulation mechanism may be more complicated in cases involving osteosarcoma patients. To confirm that lnc-SELPLG-2:1 regulates osteosarcoma via the lnc-SELPLG-2:1/hsa-miR-10a-5p /BTRC axis, the cell function of pcDNA3.1-lnc-SELPLG-2:1 was validated by transfection with hsa-miR-10a-5p mimics. Co-transfection of pcDNA3.1-lnc-SELPLG-2:1 and hsa-miR-10a-5p mimics decreased cell proliferation, migration, and invasion ability, and increased apoptosis and hsa-miR-10a-5p reversed the osteosarcoma-promoting effect of lnc-SELPLG-2:1. However, BTRC, VIM, MPP2, and MPP9 protein expression trends were consistent with these trends. Therefore, we believe that lnc-SELPLG-2:1 regulates osteosarcoma by promoting the mRNA and protein expression of BTRC via the competitive binding of hsa-miR-10a-5p.

## Conclusion

Our study provides insight into lnc-SELPLG-2:1 as a biomarker of osteosarcoma, which can be used for diagnostic and therapeutic purposes. In addition, we demonstrated that lnc-SELPLG-2:1/hsa-miR-10a-5p/BTRC is a candidate regulation pathway for lncRNA in osteosarcoma. In order to further validate the regulation mechanism, it may be necessary to determine the expression level of the targets of lnc-SELPLG-2:1 in clinical specimens. Our findings indicate that the regulation appears to be complex; thus, we suggest that more lnc-SELPLG-2:1 targets should be included in the validation process, and its mechanism of action could be better elucidated through further research.

## Data Availability

The datasets generated and/or analyzed during the current study are not publicly available due to further research is being done base on RNA sequencing but are available from the corresponding author on reasonable request.

## Abbreviations

lncRNAs: Long non-coding RNAs
AJCC: American Joint Committee on Cancer
FISH: Fluorescent in situ hybridization
